# Miasm and Contagion

**Published:** 1835

**Authors:** John L. Riddell

**Affiliations:** Adjunct Professor of Chemistry, and Lecturer on Botany in the Cincinnati Medical College


### Art. V.—Memoir on the nature of Miasm and Contagion.

*Read to the Cincinnati Medical Society, Feb. 3d,* 1836. By John L. Riddell, Adjunct Professor of Chemistry, and Lecturer on Botany in the Cincinnati Medical College.

The age in which we live is distinguished over all that have preceded it, in the advances that have been made in the physical sciences. Men seem to have become convinced, that little value can attach to abstract generalizations; and that the true mode of studying nature, is by following patiently in her footsteps, watching her silent progress, and developing her manifold and harmonious ways by experimental inquiry. Nature is limitless;—man is finite:—consequently there must be boundless fields of ever-during truth, unmarked by the footsteps of man, and unseen by the telescopic vision of research. Even within what we call the circle of human knowledge, blanks are met with, wide and dreary, and regions which seem shrouded in perennial mist. In contemplating the phenomena of nature, the mind is ever prone to fill up these vacuities with creations of its own; and then combining the real with the imaginary, the known with the unknown, to mould the whole into the form of a general system. But the experience of the world has clearly shown, that hypotheses embracing wide ranges of facts or principles, are exceedingly liable to prove more or less erroneous. They should, therefore, in all cases, be held liable to amendments, to modifications, or even to entire dismissal, upon the development of new facts making such requisitions.

Proudly as we boast of the investigating spirit of the times, very many of the theories and opinions which at this day obtain general assent, had their origin in the dark ages of science, and have been borne down to us by the current of tradition. New and relevant truths are from time to time brought to light, indicating in some instances the propriety of their total abandonment; but the fascinated minds of men are wedded to the prevalent hypotheses, and but too often, the requirements of venerable systems are mistaken for the immutable laws of nature. The novel facts are perhaps doubted, controverted, overlooked, deemed anomalous, or made to bend and accommodate themselves to the favorite theory.

The leading views I am about to offer, have been suggested at various times and by various writers. If I mistake not, however, it has always been unfashionable to believe in them, and still more so to advocate them. When the science of physiology was infinitely more vague than it is now, ere the genius of a Hunter or a Cuvier had brought to light the structure of the lower tribes of beings, or the microscope revealed its atomic worlds of animate wonders; while chemistry was yet in its feeble infancy, before the nature of the atmosphere had been investigated, or the laws of union in definite proportions determined; then might such doctrines have been considered as resting on slender probabilities. But at this time, with the multiplied results of careful observation and skilful experiment before us, it is certainly far otherwise: wherefore in venturing to stem the current of popular opinion, brooking the intolerance of some, and the ridicule of others, I feel upborne by the consciousness, that my conclusions are indicated by analogies the most clear, and facts the most incontrovertible.

I assume that the matter of contagion is of an *organized* nature, and consequently subject to the same general laws, which regulate the origin, increase, modes of existence, and duration of animal or vegetable bodies. I assume also, that the same is true of the morbific miasms which are exhaled from putrid marshes, and of the occult causes of cholera and other epidemic diseases.

In order that due weight may be assigned to the facts and arguments on which this hypothesis rests, I will first notice some of the prominent points of contrast between organized and inorganic bodies. Organized beings, animals and plants, possess forms more or less rounded, and never exceed in size certain definite dimensions:—mineral or inorganic bodies as a class, have no general determinate form, unless it be the angular one assumed by them in the crystalline condition, and in respect to their size, there are seldom any assignable limits. Organized bodies are made up essentially of the ultimate elements, oxygen, hydrogen, and carbon; or of oxygen, hydrogen, carbon, and nitrogen; in all cases united in a most complicated manner. The composition of individual minerals is always characterized by definiteness and comparative simplicity, though as a class, they embrace all known elementary bodies.

Minerals increase in size by the external addition of particles similar to their own; and the causes which elaborate and supply these particles, are wholly independent of the bodies which they tend to augment. Animals and vegetables possess within themselves a power of elaboration; they consequently increase in size by the internal assimilation of foreign particles. In all organic structures of the more highly developed types, there exist harmonious and dependent series of vessels, tissues, and organs, which concur in effecting this end. Of this internal conformation, minerals are wholly devoid. The same causes which develope and perfect the organic structure, ultimately put a limit to the duration of individual beings;—whereas all brute matter is endued with the negative attributes and conditions requisite to insure endless perpetuity. Though a finite period of existence is allotted to individual plants and animals, it is believed by many that nature has fixed no bounds to the duration of species. Cuvier and Roget recognize this opinion as a settled point in physiology;—yet I confess it is more consonant with what seems to me the general ways of nature, to suppose that particular species or kinds lose their identity in process of time, by giving origin to modified kinds or varieties; which in turn may long wear the aspect of confirmed species, and ultimately, in like manner, give way to their successors. However this may be, there is certainly no assignable limit to the reproduction, succession, and continuation of organized beings.

I claim for the corpuscles which cause malarious and infectious maladies, trans-microscopic as they are, the humble rank of belonging near the lower confines of organic nature. Perhaps they hold nearly the same grade in respect to animate and sentient beings, which the more simple and minute of the *Fungi* and *Algæ* do to the more perfect tribes of vegetables. Inconceivably minute as they doubtless are, they must yet possess a share of vitality, because like other animals and plants which come under our observation, they have the power of propagating and extending themselves indefinitely. With a view of throwing light by analogy on the possible habitudes of miasmatic molecules, and demonstrating at the same time the strange and almost incredible capabilities of animal life, I shall adduce a few well authenticated observations on the nature and habits of infusory animalcules:—though in honesty I do not imagine there exist very close resemblances between the animalcules which the microscope displays to our view, and the miasmatic corpuscles which elude its cognizance.

*Infusoria* is the name of an obscure class of the animal kingdom, characterized more by the minuteness of the beings it embraces, than by any particular features or structure which they possess in common. This appellation was proposed by the Danish naturalist, Muller, distinguished for his acquaintance with this department of zoology, on account of the fact, that myriads of these little creatures always make their appearance in fermenting infusions of animal or vegetable matter. They are, by no means, however, confined to such infusions, being frequently met with on the leaden tiles of houses, in putrefying sores and depraved humors of man and beast, and in one, at least, of the healthy animal fluids. Countless multitudes of a parti-colored, eel-like animalcule, (*Vibrio tritici*,) exist in the pulverulent substance within the glumes of blighted wheat. Dr. Lindley, of the University of London, says, (First Principle of Botany, 329,) that the granules of pollen, (the fecundating dust, elaborated by the flowers of plants,) inclose a mucuous substance, in which is contained an infinite number of exceedingly minute molecular bodies, having a power of active motion. It is now generally believed, that animalcules do not occur in pure fountain water, nor in many of the healthy animal fluids. To account for the occurrence of these animated atoms in particular situations, baffles the most untiring ingenuity of research. Spallanzani made some instructive experiments with a view of determining the condition preclusive of, as well as those most favorable to their production. He placed portions of the same vegetable infusion in different glass vessels. Some were left exposed to the air, others slightly covered, and others hermetically sealed. He found, subsequently, the greatest numbers of infusoria, in those vessels which were most freely exposed, yet they occurred in the infusions hermetically sealed. Boiling the infusion, though it reduced their numbers, did not entirely prevent their appearing. In one instance he boiled the infusion for an hour, and then hermetically sealed it: after the lapse of twenty-five days, a a few of the smaller kinds of animalcules had been developed. They readily appear under reduced pressure, where the air will support only thirteen inches of mercury.

Some species of animalcules have the power of enduring great changes of temperature in the medium containing them, with apparent impunity. The *Vermiculi tauri*, which inhabit a medium of 103 deg. Fahr., support 5 deg. without death, and retain their vivacity a long time at 32 deg., the freezing point of water. The same beings are not destroyed by raising the temperature to 133 deg. The native medium of the *Vermiculi hominis*, is usually maintained at 98 deg. These vermiculi retain vitality between 32 and 131 deg. The common infusory animalcules are capable of brooking a range of temperature, from the freezing of water, to near 110 deg.

Collateral facts, drawn from the vegetable kingdom, show, in a manner equally striking, the great power sometimes possessed by organic nature, of enduring extremes of heat or cold. Immense fields of scarlet snow, are sometimes presented to the traveller, in the intensely cold regions of the northern frigid zone. Investigation has shown, that the color depends upon millions of microscopic plants, belonging to the order *Fungi*. The *Ulva thermalis*, a plant of the order *Algæ*, and the *Limneus pereger*, a fresh water shell, are found in Gastein thermal springs, where the constant temperature is 117 deg. (De la Beche, p. 18.)

The most mysterious circumstance in the natural history of the infusoria, is the susceptibility which some of them possess, of remaining an indefinitely long time in a perfectly dry, and seemingly lifeless condition.

The same prerogative is enjoyed, to a certain extent, by many vegetable seeds, and by certain worms of the order

*Annelida;* but the resurrection of animalcules, taking place more rapidly, is far more striking to the observer. "The *Rotifer redivivus*, or wheel animalcule, which was first observed by Lewenhoeck, and was afterwards rendered celebrated by the experiments made upon it by Spallanzani, can live only in water, and is commonly found in that which has remained stagnant for some time in the gutters of houses. But it may be deprived of this fluid, and reduced to perfect dryness, so that all the functions of life may be completely suspended, yet without the destruction of the vital principle; for this atom of dust, after remaining for years in a dry state, may be revived in a few minutes by being supplied with water. This alternate suspension and restoration of life may be repeated, without apparent injury to the animalcule, for a great number of times. Similar phenomena are presented by the *Vibrio tritici*, an eel-like animalcule, which infests diseased wheat, and which, when dried, appears in the form of a fine powder. On being moistened it soon resumes its living and active state.

The *Gordius aquaticus*, or hair worm, which inhabits stagnant pools, and which remains in a dry and apparently lifeless state when the pond is evaporated, will, in like manner, revive in a very short time, on being again immersed in water. The same phenomenon is exhibited by the *Filaria*, a thread-like parasitic worm, infesting the cornea of the horse." (Roget. Physiology. I. 58.)

In the Edinburgh Encyclopedia, article Animalcule, it is stated, that the wheel animalcules have been thus resuscitated from a state of dormant vitality, as many as seventeen times in succession, and that the presence of sand is necessary in the fluid, or they will not revive. That when active, 113 deg. is sufficient to kill them, but when dry, the vital principle is not destroyed unless the temperature be raised to 158 deg.; or if, while in this condition, they be exposed to the intense cold of — 11 deg., they may be subsequently revived. Strong camphoric and terebinthinate odors prevents reanimation.

In no other department of animated nature, are we presented with such strange and anomalous modes of reproduction. Many of the globular *Monades* and *Vorticellæ*, increase by spontaneous and equal division. The living globule will at first appear as if encircled by an equatorial band, which will continue to be drawn more and more tight, until a complete separation occurs; each portion being an independent monad, which in turn is bisected like its parent. In this manner, a mysterious multiplication goes on indefinitely. The *Monas uva* consists of four or five corpuscles in a cluster, by the spontaneous separation of which, the species is propagated. Errhenberg has determined that the smaller monads are near one twenty-four thousandth of an inch in diameter; and he has estimated that there are 500,000,000 of them in the space of a cubic line, or drop of liquid which he examined.

The *Volvox globator* consists of a spherical, membranous sac, filled with liquid, in which float many more diminutive globules like itself. These have precisely the same structure with the enveloping membrane, even to containing within them a series of still minuter spherules. Observers have thus seen the fifth generation in the same individual. The parent always ends its own existence in giving birth to its progeny. The *Gonium pectorale* has an angular, flattened body, containing sixteen corpuscles, which subsequently become distinct animalcules like those in the volvox. A curious being, provided with a beak, was observed by M. Bonnet, in an infusion of hemp, which fixing itself to some solid substance, assumes a spherical form, and rotates irregularly until it bursts into four animalcules. Spallanzani took pains to isolate the egg of an animalcule on a watch glass: in a short time it was developed, became mature, and produced eggs like the one from which it sprung; and these likewise followed the same habits.

It is indeed astonishing to observe how short a time is sufficient, in some instances, to bring these beings to full maturity. An infusion of beet yields a species that increases by detaching obliquely a small piece of its own substance, which, after the lapse of a single day, is also capable of propagating. Strange as this may appear, the instance is less remarkable than that of the *Vorticella ramosa*, which exercises the power of reproduction, within a few hours only, after having been itself ushered into existence.

So far as investigation has been carried, this proposition is fairly established: that animal or vegetable food is essential to every subject of the animal kingdom. It must therefore follow, if the proposition be universally true, that the *Infusoria* feed on organic substances; and it is probable, that many of them subsist on corpuscles more minute than themselves. Some of them are known, indeed to be carniverous. To adduce an instance, Goeze has seen the *Trichoda cimex*, a bristly, microscopic creature, of an oval form, seize upon and devour the lesser animalcules with great voraciousness.

A belief in the existence of miasmata, or morbific agents in the atmosphere, has long been entertained. When the general constitution of common air was discovered and announced to the world, it was expected that a prolific source of maladies would be found in the varying proportions of its ingredients; but repeated and careful analyses have demonstrated, that the relative quantities of oygen and nitrogen are found to be nearly constant, whether the air be taken from the infected wards of a crowded hospital, from the most pestilential marsh, or from the most pleasant and healthy situation. True, the quantity of carbonic acid in atmospheric air, varies at times from one per cent. to one in a thousand; but these variations seem to have no connexion with the prevalence or production of disease. The amount of miasmatic matter diffused through infected air, must be almost inappreciably small. M. Boussingault, of Lyon, has lately made a series of most careful experiments on the composition of air, procured from highly malarious districts. He has determined the presence of hydrogen, not existing as a constituent of water, to an extent by weight, varying from three to eight millionths of the air examined. He presumes, from other important experiments, that it was a part of something of an organic nature.

In these instances, portions of air were submitted to trial, which, though they contained less than a hundred thousandth part of any thing that could be called miasm, were nevertheless sufficiently imbued with the germs of disease to give rise to the most severe intermittent fevers. So, likewise, the remote cause of cholera may lurk, unappreciated, in the atmosphere. It is well known, that a quantity of contagion, as the virus of small pox, altogether too minute to affect the most delicate balance, is sufficient to develope a disease in the human system, which may propagate itself to an indefinite extent.

A grand and most decisive argument, relative to the probable nature of infectious miasms, may be drawn from the established laws of chemical action and combination. Multiplied researches have demonstrated, that when elementary or compound bodies chemically combine, it is invariably in certain fixed and definite proportions; thus, 108 parts of silver unite with 8 parts of oxygen; 40 parts of potassium with 8, and also with 16 parts of oxygen; 40 parts of sulphuric acid combine with 48 of potash. Consequently, substances, in producing chemical effects, lose *pari passu* the affection or power which enables them to do it: in the same manner as a moving body parts with its momentum in giving motion to other bodies. Suppose I endure, for a while, the action of a drachm of the concentrated solution of caustic potash, on the palm of my hand. The presence of the alkali will cause the decomposition of the dermoid tissues, the elements of which, will be converted into oleic, margaric, and stearic acids, that immediately combine with, and neutralize the causticty of the potash: and thus, at length, the potash will have a limit put to its action, its place being supplied by the various soap-like salts to which it gives origin, and of whose composition it forms a part.

It is after this wise, that the action of every possible dead substance must have a definite limit. A small quantity of such an agent produces only a comparatively small effect, while a large quantity produces an effect correspondingly large. The unpleasant ulcer which would follow the application of caustic potash to the hand, could not be communicated to other persons by contact;—the morbific cause would not in the slightest degree possess the power of self-propagation.

There is, then, this wide difference between contagious and ordinary poisons, that in the one, the extent of the effect depends wholly upon the quantity of material employed: in the other, the smallest possible quantity is adequate to the production of the greatest possible effect; an effect which may be felt, as in the case of small pox, by millions of human beings. The chemist has power to produce all mineral or inorganic combinations at pleasure, though unable to imitate the more complex results of vital action. Now, if the causes of pestilence be inorganic, why has the virus of small pox, the miasm of cholera, or of any other pestilential malady never chanced to originate in chemical laboratories?

Must there not then, be a degree of vitality resident in the matter of contagion? Is there not a perfect analogy between its unlimited propagation, and the unlimited propagation of animal or vegetable species? What brute force, let me ask, within the compass of inorganic nature, acts unexpended? and where may we find this high prerogative, except as a mysterious endowment of vitality?

The virus of many contagious diseases may be kept in a dry condition for a very long time, being still capable of exciting disease by inoculation. How perfectly similar this is, to the wonderful resurrection of which the dry and shapeless remnants of certain animalcules are capable; and how totally inexplicable on any other hypothesis.

Miasmatic poisons, when applied to the animal system, generally require several days, before the obvious development of any effect. This time, called the latent period, affords a strong argument in favor of the organized nature of the poison; for ordinary poisons never delay their action so long: whereas, if contagion consists of living corpuscles, like the ova of insects or the germs of plants, they would naturally require time for their development and multiplication.

The interesting experiments of Moscati and Boussingault, have shown in my opinion beyond a doubt, that organic matter exists in extremely small quantities, in the noxious air that hovers over marshes. Moscati, a learned Italian, many years ago, suspended in the air, over the rice grounds of Tuscany, a globular glass vessel, filled with ice. An abundant deposition of dew took place upon its surface, which, when collected, appeared at first to be pure and limpid water. There was soon, however, an appearance of little flakes, "possessed of properties peculiar to animalized matters, and, finally, at the end of some days, the liquid putrified completely."

M. Boussingault, in a memoir read to the French academy of Sciences, in August, 1834, details some striking experiments made by him at Cartago, South America. "A little after sunset," says he, "I placed two watch glasses on a table standing in the middle of a swampy meadow. In one of the glasses I poured hot distilled water, in order to wet its surface, and, at the same time, to communicate to it a temperature higher than that of the air. The cold glass, its temperature being lowered by the nocturnal radiation, was soon covered with an abundant dew. The warm glass could not evidently condense dew. On adding a drop of distilled sulphuric acid to each glass, and evaporating to dryness with the heat of an alcahol lamp, I always saw a trace of carbonaceous matter adhering to the glass in which the dew had been deposited, while the glass which had not condensed dew, was perfectly clean after the volatilization of the acid." I may here add, that strong sulphuric acid decomposes and blackens organic matters, in consequence of its affinity for water; causing the oxygen and hydrogen of those substances to unite in the production of water, and leaving the carbon in the condition of finely divided charaoal, which accounts for the occurrence of the black color. The same writer further says, that he soon experienced upon himself the effects of the miasm, whose presence he was endeavoring to prove: for he was attacked with a fever which forced him to interrupt his researches.

(Concluded under the head of Original Intelligence.)

